# *Streptococcus canis* Are a Single Population Infecting Multiple Animal Hosts Despite the Diversity of the Universally Present M-Like Protein SCM

**DOI:** 10.3389/fmicb.2019.00631

**Published:** 2019-03-29

**Authors:** Marcos D. Pinho, Geoffrey Foster, Constança Pomba, Miguel P. Machado, Johanna L. Baily, Thijs Kuiken, José Melo-Cristino, Mário Ramirez, The Portuguese Group for the Study of Streptococcal Infections, Teresa Vaz

**Affiliations:** (Centro Hospitalar do Barlavento Algarvio); (Centro Hospitalar de Entre Douro e Vouga); (Centro Hospitalar de Leiria); (Centro Hospitalar de Vila Nova de Gaia/Espinho) (Centro Hospitalar do Alto Ave); (Centro Hospitalar do Porto); (Centro Hospitalar da Póvoa do Varzim/Vila do Conde); (Hospital Central do Funchal); (Centro Hospitalar de Lisboa Central); (Centro Hospitalar Lisboa Norte); (Centro Hospitalar Lisboa Ocidental); (Centro Hospitalar do Baixo Vouga); (Hospital de Vila Real); (Hospitais da Universidade de Coimbra); (Hospital de Cascais); (Hospital de São João Porto); (Hospital de Braga); (Hospital de Santa Luzia Elvas); (Hospital dos SAMS Lisboa); (Hospital Dr. Fernando da Fonseca Amadora/Sintra); (Hospital do Espírito Santo Évora); (Hospital Garcia de Orta Almada); (Hospital Pedro Hispano Matosinhos); (Unidade Local de Saúde do Baixo Alentejo Beja); ^1^Instituto de Microbiologia, Instituto de Medicina Molecular, Faculdade de Medicina, Universidade de Lisboa, Lisbon, Portugal; ^2^SRUC Veterinary Services, Inverness, United Kingdom; ^3^Faculdade de Medicina Veterinária, Universidade de Lisboa, Lisbon, Portugal; ^4^Moredun Research Institute, Pentlands Science Park, Edinburgh, United Kingdom; ^5^Department of Viroscience, Erasmus Medical Center, Rotterdam, Netherlands

**Keywords:** *Streptococcus canis*, multilocus sequence typing, M-like protein (SCM) gene, wildlife, genome

## Abstract

*Streptococcus canis* is an animal pathogen which occasionally causes infections in humans. The *S. canis* M-like protein (SCM) encoded by the *scm* gene, is its best characterized virulence factor but previous studies suggested it could be absent in a substantial fraction of isolates. We studied the distribution and variability of the *scm* gene in 188 *S. canis* isolates recovered from companion animals (*n* = 152), wild animal species (*n* = 20), and humans (*n* = 14). Multilocus sequence typing, including the first characterization of wildlife isolates, showed that the same lineages are present in all animal hosts, raising the possibility of extensive circulation between species. Whole-genome analysis revealed that *emm*-like genes found previously in *S. canis* correspond to divergent *scm* genes, indicating that what was previously believed to correspond to two genes is in fact the same *scm* locus. We designed primers allowing for the first time the successful amplification of the *scm* gene in all isolates. Analysis of the *scm* sequences identified 12 distinct types, which could be divided into two clusters: group I (76%, *n* = 142) and group II (24%, *n* = 46) sharing little sequence similarity. The predicted group I SCM showed extensive similarity with each other outside of the *N*-terminal hypervariable region and a conserved IgG binding domain. This domain was absent from group II SCM variants found in isolates previously thought to lack the *scm* gene, which also showed greater amino acid variability. Further studies are necessary to elucidate the possible host interacting partners of the group II SCM variants and their role in virulence.

## Introduction

*Streptococcus canis* is a beta-hemolytic Lancefield group G streptococcus which colonizes the skin, the upper respiratory tract and the reproductive tract of dogs and cats (Devriese et al., 1986; Lyskova et al., 2007; Timoney et al., 2017). *S. canis* is also an important pathogen of these species, causing skin, and genitourinary tract infections, otitis externa, pneumonia, endocarditis, septic arthritis, septicemia, necrotizing fasciitis, and streptococcal toxic shock syndrome (DeWinter et al., 1999; Lyskova et al., 2007; Morrow et al., 2016). The isolation of *S. canis* from other animals has been documented, including livestock, in which it is known to cause clinical and subclinical bovine mastitis (Hassan et al., 2005), and various wild animal species, including minks (Chalmers et al., 2015) and feral cats (Hariharan et al., 2011), but also aquatic mammals such as pinnipeds (Castinel et al., 2007; Seguel et al., 2018) and otters (Simpson, 2006). *S. canis* is a well-recognized zoonotic agent, with a growing number of studies reporting the isolation of *S. canis* from cases of skin and soft tissue infections, bacteremia and endocarditis in humans (Whatmore et al., 2001; Pinho et al., 2013; Amsallem et al., 2014; Taniyama et al., 2017). Although it has been shown that the same *S. canis* lineages are found in companion animals, livestock and humans (Richards et al., 2012; Pinho et al., 2013), genotypic characterization of *S. canis* isolates from wild animals is limited.

Genomic information showed that *S. canis* is related to beta-hemolytic *Streptococcus pyogenes* (group A streptococcus [GAS]) and *Streptococcus dysgalactiae* subsp. *equisimilis* (SDE), both human pathogens with which *S. canis* shares many putative virulence factors (Richards et al., 2012). The M protein, encoded by the *emm* gene, is one of the main virulence factors of both GAS and SDE (Cunningham, 2000; Brandt and Spellerberg, 2009), but application of *emm* typing – a technique based on amplification and sequencing of a segment of the *emm* gene encoding the *N*-terminal hypervariable portion of the protein – revealed that most *S. canis* isolates were non-typeable (Ahmad et al., 2009; Pinho et al., 2013). Subsequently, an M-like protein was identified in the *S. canis* genome (termed SCM for *S. canis* M-like protein; Fulde et al., 2011), encoded by the *scm* gene, which is now the best characterized of the *S. canis* virulence factors.

*Streptococcus canis* M-like protein was first described by Yang et al. (2010) named SPASc by the authors, who showed a protective response in mice after passive immunization and opsonization with specific antiserum. It was shown that SCM binds both plasminogen and IgG from various animal species, including humans, through domains present in the *N*-terminus and central part of the mature protein, respectively (Fulde et al., 2011; Bergmann et al., 2017). However, these studies also suggested that up to one third of the *S. canis* isolates lack the *scm* gene, since the authors correlated failure in amplifying *scm* by PCR with weak plasminogen and immunoglobulin-binding capacity (Fulde et al., 2011, 2013; Bergmann et al., 2017). The *scm* gene was detected in *S. canis* isolates from distinct hosts, including dogs (Fulde et al., 2013), cats (Timoney et al., 2017), cows (Richards et al., 2012) and humans (Fulde et al., 2011; Taniyama et al., 2017), and a recent study showed that allelic variants of *scm* found among cat isolates in the United States could be classified into four distinct types (Timoney et al., 2017). However, the correspondence between the *scm* and the *emm* locus remains uncertain since the *scm* sequences described to date (Richards et al., 2012; Taniyama et al., 2017;Timoney et al., 2017) are distinct from the *emm* sequences derived from *S. canis* isolates (Ahmad et al., 2009; Pinho et al., 2013) and previous studies have applied PCR protocols targeting specifically either *scm* or *emm* with no information regarding their genomic context.

The goal of the present study was to determine the distribution of *scm* in *S. canis* isolates recovered from different hosts, including wild animals. The draft genomic sequence of an *emm-*typeable *S. canis* isolate (Pinho et al., 2013) in which the *scm* PCR amplification failed was determined, with the aim of clarifying the correspondence between *scm* and *emm* and establishing a protocol to allow typing of all *S. canis* isolates. Multilocus sequence typing (MLST) was used to determine the genetic diversity of the collection and to allow a comparison between *S. canis* isolates recovered from wild animal species, companion animals and humans.

## Materials and Methods

### Bacterial Isolates

A collection of 188 *S. canis* isolates was analyzed. Ninety-five *S. canis* isolates were recovered from dogs (*n* = 75) and wild animal species (*n* = 20), including seals (*n* = 11), otters (*n* = 6), badgers (*n* = 2) and a fox (*n* = 1). Seal species included gray seals (*Halichoerus grypus*, *n* = 6 isolates), common seals (*Phoca vitulina, n = 2*) and Mediterranean monk seals (*Monachus monachus, n* = 2), while in one case the seal species was not recorded. Isolates from wild animals were recovered during necropsies of carcasses found in the wild, while those of companion animals were recovered within the normal workup for the diagnosis of suspected infections. No samples were obtained specifically for this study. All isolates were recovered in Scotland from 1993 to 2014, however, the two isolates from Mediterranean monk seals were cultured in Scotland, following necropsies in the Netherlands. All isolates were presumptively identified by the contributing laboratories using biochemical tests. For comparison purposes and to maximize the diversity of the *S. canis* isolates on which the distribution of the *scm* gene was to be analyzed, an additional set of 93 *S. canis* isolates was included in the study: 7 isolates recovered from human infections in Portugal, from 2011 to 2017, from blood (*n* = 2), pus (*n* = 2), sputum, urine and vaginal exudate (*n* = 1 each); and 86 isolates previously characterized by MLST (Pinho et al., 2013), recovered in Portugal, Germany and Italy, from dogs, cats, a horse and humans. The study was approved by the Institutional Review Board of the Centro Académico de Medicina de Lisboa. These were considered surveillance activities and were exempt from informed consent. All methods were performed in accordance with the relevant guidelines and regulations. The data and isolates were de-identified so that these were irretrievably unlinked to an identifiable person. Detailed information on all isolates included in the study is provided in Supplementary Table 1.

### Hemolysis and Lancefield Grouping

Beta-hemolysis and colony size were confirmed in tryptic soy agar (Oxoid, Hampshire, United Kingdom) supplemented with 5% (vol/vol) defibrinated sheep blood, after overnight incubation at 37°C. The Lancefield group was confirmed by a commercial latex agglutination technique (Streptococcal Grouping Kit, Oxoid, Basingstoke, United Kingdom).

### MLST Analysis

*Streptococcus canis* isolates were characterized using the MLST scheme available for *S. canis*^1^ (Pinho et al., 2013). The PCR amplification and sequencing for some of the loci were optimized by designing novel primers based on *S. canis* genomes (Supplementary Table 2). Unique sequences at each locus were assigned allele numbers. The combination of the seven allele numbers for each isolate was used to define sequence types (STs). An expansion of the goeBURST algorithm implemented in PHYLOViZ (Nascimento et al., 2017) was used to generate a minimum-spanning-tree-like reflecting possible relationships between *S. canis* STs. Clonal complexes were defined at the single-locus variant level (CC_SLV_).

### Whole Genome Sequencing

The genome of *S. canis* isolate FMV2238.02 was sequenced using Illumina MiSeq. This isolate was recovered from a dog ear exudate in 2002 in Portugal, belongs to MLST ST1 and has the *emm* type *stG1389* (Pinho et al., 2013).

Whole-genome sequencing library was prepared using paired-end Nextera^®^XT DNA Library Prep Kit, Index Kit v2 (Illumina©, San Diego, CA, United States) and sequenced on Illumina MiSeq^®^system (Illumina©) using MiSeq^®^Reagent Kit v2 Kit (500 cycles) at the Genomics Unit of Instituto Gulbenkian de Ciência (Oeiras, Portugal). The quality of the 250 bp paired-end reads obtained was assessed with INNUca pipeline,^2^ which also assembles and curates the bacterial genomes. INNUca v3.1 was run using Docker image “ummidock/innuca:3.1”^3^ using a predicted genome size of 2.1 Mb. Briefly, reads quality were checked with FastQC^4^ and cleaned using Trimmomatic (Bolger et al., 2014). *De novo* assembly was performed using SPAdes (Bankevich et al., 2012) and subsequently polished using Pilon (Walker et al., 2014). Genomes were annotated using Prokka pipeline v1.12 (Seemann, 2014) using Docker image “ummidock/prokka:1.12”^5^. SignalP v4.1 (Petersen et al., 2011) was used to find signal peptide features and RNAmmer v1.2 (Lagesen et al., 2007) was used to find ribosomal RNA features, both were externally provided to the Docker container. Prokka was run using the following parameters: –addgenes –usegenus –rfam –rnammer –gram pos –increment 10 –mincontiglen 1 –gcode 11 –kingdom Bacteria –genus Streptococcus –species canis.

The locus corresponding to *emm stG1389* was identified and the flanking regions were extracted and aligned with the corresponding regions where *scm* was found in the 3 publicly available *S. canis* genomes,^6^ namely strains FSL Z3-227 (GenBank accession number NZ_AIDX01000001), G361 (GenBank accession number NZ_NMRV01000001) and TA4 (GenBank accession number NZ_BEWZ01000010).

### *emm* Typing and *scm* Amplification

Characterization of the *S. canis* isolates by *emm* typing was carried out with primers (emm1 and emm2) and conditions available at https://www.cdc.gov/streplab/groupa-strep/emm-typing-protocol.html. The *scm* gene was initially amplified by using the primers all-canis_fwd and all-canis_rev described by Fulde et al. (2011). To enable *scm* amplification and sequencing of the whole *scm* gene in all *S. canis* isolates, new primers (Sc_Mprot_F1 and Sc_Mprot_R1) were designed (based on the whole genome comparisons) targeting the *scm*/*emm* flanking genes, resulting in amplification of an 1825bp fragment in *S. canis* isolate FMV2238.02 (Supplementary Figure 1 and Supplementary Table 2). Briefly, 3 μl of template DNA was added to the PCR mixture containing 1U GoTaq G2 Flexi DNA polymerase (Promega, Madison, WI, United States), 1X Green GoTaq Flexi Buffer (Promega, Madison, WI, United States), 1.5 mM MgCl_2_ (Promega, Madison, WI, United States), 200 μM deoxynucleoside triphosphates (Thermo Fisher Scientific, Waltham, MA, United States) and 0.4 μM primers, in a final volume of 50 μl. The PCR conditions were adapted from the *emm* typing protocol, as follows: 94°C for 1 min; 10 cycles of 94°C for 15 s, 46°C for 30 s and 72°C for 1 min 15 s; 25 cycles of 94°C for 15 s, 46°C for 30 s and 72°C for 1 min 15 s with a 10 s increment for each of the subsequent 24 cycles; final extension at 72°C for 10 min. All PCR reactions were conducted in a Biometra T gradient thermocycler (Goettingen, Germany). Sequencing of the PCR products was carried with the primers used for amplification, plus primers Sc_Mprot_F2 and Sc_Mprot_R2 which have target sequences inside the amplified fragment.

### Analysis of *scm* Sequences

Geneious (version 8.1.9, Biomatters, Auckland, New Zealand) was used to identify the putative *scm* open reading frames (ORFs). Sequences were aligned with MUSCLE and MEGA (version 7.0.18) (Kumar et al., 2016) was used to construct a neighbor-joining (NJ) tree of *scm* alleles by using the Kimura two-parameter substitution model. Branch support was tested by 1,000 replicate bootstrap tests in each analysis. The sequences of the *scm* gene extracted from the *S. canis* genomes and those previously reported by Timoney et al. (2017) were included in the analysis for comparison. The *scm* types were assigned based on the full *scm* sequence alignment and named following the designations of the allelic variants identified previously (Timoney et al., 2017). The *scm* ORFs were translated into amino acid sequence and the SignalP 4.1 server^7^ was used to identify the signal peptides. The SCM amino acid sequence derived from the genome of isolate G361, a strain included in the studies that described the IgG and plasminogen-binding regions of SCM (Fulde et al., 2011; Bergmann et al., 2017), was used as reference to identify these regions in *S. canis* isolates. Search for protein domains was carried out with InterPro version 69.0^8^.

### Statistical Analysis

The diversity and congruence of MLST STs and *scm* types were quantitatively evaluated by calculating Simpson’s indices of diversity (SID) (Carriço et al., 2006) and adjusted Wallace (AW) coefficients (Severiano et al., 2011) with 95% confidence intervals (CIs). Calculations were performed using the Comparing Partitions website^9^. The Fisher exact test was used to explore differences between the distribution of the two *scm* groups between hosts.

## Results

### Characteristics and Clonality of *S. canis* Isolates

**FIGURE 1:**
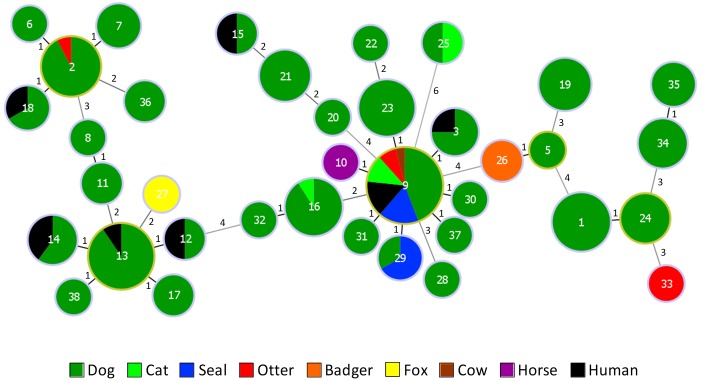
goeBURST diagram of *S. canis* isolates recovered from animal and human infections. The diagram includes the 188 isolates studied plus the 3 *S. canis* isolates with publicly available genomes (from which MLST data was extracted). Numbers inside the circles identify the ST and numbers near the lines indicate the number of alleles different between the two connected STs. The size of each circle is proportional to the number of isolates in a logarithmic scale. The number of isolates with the same characteristic is proportional to the respective color. Putative CC founders are identified by an outer light green circle and correspond to the STs with the higher number of SLVs.

All isolates were beta-hemolytic and carried the Lancefield group G polysaccharide, as is characteristic of *S. canis*. The 188 *S. canis* isolates belonged to 37 STs (SID ± 95% CI, 0.899 ± 0.030), 13 of which were novel (STs 26 to 38, *n* = 23 isolates). Eight CC_SLV_ were identified (Figure 1). The 95 isolates from the Scottish collection belonged to 24 STs (SID ± 95% CI, 0.899 ± 0.039), a similar diversity to the one observed for the remaining *S. canis* isolates included in the analysis (24 STs, SID ± 95% CI, 0.887 ± 0.042). Dog isolates recovered in Scotland and those recovered elsewhere shared 10 STs present in the main CC_SLV_. Wildlife isolates belonged to 6 different STs, three of which (STs 2, 9, and 29) were identical to those found among dog isolates, while ST26 (two badgers), ST27 (one fox), and ST33 (one otter) were, respectively SLV, double-locus variants and triple-locus variants of dog isolates. Seal isolates belonged to a single CC_SLV_, presenting either ST9 (*n* = 9) or ST29 (the two Mediterranean monk seals sampled in the Netherlands). On the other hand, *S. canis* isolates from otters belonged to three STs: ST9 (*n* = 4), ST2 and ST33 (one isolate each).

### Distribution of *emm* and *sc*m

By *emm*-typing we could only type 22 of the 188 isolates (11.7%). *stG1389* (*n* = 18), *stG1451* (*n* = 2) and *stG663* (*n* = 2) were the three *emm* types found. On the other hand, 142 isolates (75.5%) were positive for the *scm* gene in the PCR using the all-canis primers (Fulde et al., 2011). The 46 (24.5%) *S. canis* isolates that were *scm* negative in this PCR included the 22 isolates with an assigned *emm* type and 24 isolates negative in both PCRs.

### Identification of *scm* in the Genome of an *stG1389* Isolate

To clarify if the amplification obtained in the *emm* typing protocol corresponded to the *scm* locus, the genome of *S. canis* isolate FMV2238.02 (ST1, *emm* type *stG1389*) was sequenced. The *stG1389* sequence in this isolate was found to correspond to an *emm-*like gene flanked by genes encoding a putative *trans*-acting positive regulator (upstream) and a bifunctional enzyme (downstream). Alignment with the corresponding regions in the three available *S. canis* genomes, showed that while the *stG1389 emm-*like gene had less than 58% sequence identity to the *scm* genes of the other strains, the flanking genes have more than 96% (*trans*-acting positive regulator) and 97% (bifunctional enzyme) sequence identity in the 4 strains, confirming that the *emm*-like and *scm* genes of these strains are present in the same genomic context and therefore correspond to the same locus, henceforth named *scm* (an example of the alignment with strain FSL Z3-227 is provided in Supplementary Figure 1). PCR amplification and sequencing using primers targeting the extremities of the two flanking genes (primer pair Sc_MProt_F1/R1) confirmed the presence of an *scm* gene in this location in all 188 *S. canis* isolates (fragment size ranging from approximately 1.5 to 2 Kb), including those that did not yield a PCR product with either the *emm* typing protocol or with the all-canis primers. The extensive sequence variation in the *scm* regions where the emm1/2 and all-canis_fwd/rev primers bind could potentially justify the failure of the amplification with these primers in some isolates (Supplementary Figure 1).

### Correlation of *scm* Types With MLST STs

**FIGURE 2:**
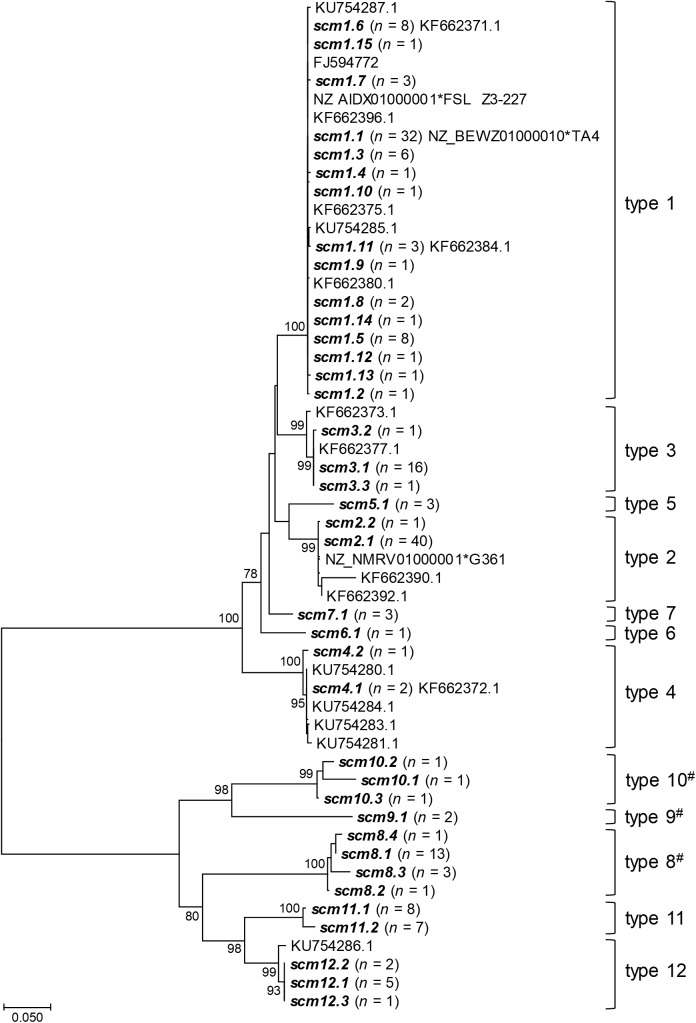
Neighbor-joining tree of *scm* alleles found among *S. canis* isolates. The *scm* alleles found in the current study are shown in bold and the numbers of isolates presenting each allele are indicated in brackets. Sequences from other studies are identified by their GenBank accession number and include those previously described by Timoney et al. (2017), the original *scm* sequence (FJ594772) described by Yang et al. (2010) and those extracted from *S. canis* genomes (indicated by an asterisk following the accession number and the name of the strain). *scm* alleles 1.16 to 1.18 have premature stop codons and were not included in the tree. Branches supported in the bootstrap test (1,000 replicates) by > 75%, have the values shown next to the branches. The tree is drawn to scale, with branch lengths being in the same units as those of the evolutionary distances used to infer the phylogenetic tree. The evolutionary distances were computed by the Kimura two-parameter method and are in units of number of base substitutions per site. #indicates *scm* types that correspond to previously identified *emm* types, namely *stG1389* (*scm8*)*, stg1451* (*scm9*), and *stG663* (*scm10*).

There were 41 distinct *scm* alleles among the 188 *S. canis* isolates studied, which could be divided into 12 *scm* types (SID ± 95% CI, 0.776 ± 0.042; Table 1). Types 1 (*n* = 73, 38.8%) and 2 (*n* = 41, 21.8%) were the dominant *scm* types, together accounting for around 60% of the isolates. The NJ tree of the *scm* alleles found (Figure 2) shows two main clusters, one including s*cm* types 1 to 7 (group I *scm* alleles), present in 75.5% of the isolates (*n* = 142), and the other including *scm* types 8 to 12 (group II *scm* alleles), found in 24.5% of the isolates (*n* = 46). While group I included all the isolates that were *scm*-typeable with the all-canis primers previously described (Fulde et al., 2011), group II included the non-typeable isolates, some of which had an *emm* type assigned in the *emm* typing protocol (*scm* types 8, 9, and 10, corresponding to *emm* types *stG1389*, *stG1451*, and *stG663*, respectively). Group II *scm* alleles were found among 42 dog isolates, 2 badgers, 1 fox, and 1 human isolate (Table 1), with no differences in the distribution of the two groups between hosts.

**Table 1:** Characteristics of the 188 S. canis isolates.

*scm* type	*emm* type*^a^*	*scm* PCR*^b^*	No. of *scm* alleles	Hosts (No. of isolates)	No. of isolates (%)
1	NT	positive	18	Dog (42), seal (11), human (8), cat (6), otter (4), cow (1), horse (1)	73 (38.8)
2	NT	positive	2	Dog (36), human (4), cat (1)	41 (21.8)
3	NT	positive	3	Dog (16), otter (1), human (1)	18 (9.6)
4	NT	positive	2	Dog (1), cat (1), otter (1)	3 (1.6)
5	NT	positive	1	Dog (3)	3 (1.6)
6	NT	positive	1	Dog (1)	1 (0.5)
7	NT	positive	1	Dog (3)	3 (1.6)
8	*stG1389*	negative	4	Dog (17), fox (1)	18 (9.6)
9	*stG1451*	negative	1	Dog (2)	2 (1.1)
10	*stG663*	negative	3	Badger (2), dog (1)	3 (1.6)
11	NT	negative	2	Dog (14), human (1)	15 (8.0)
12	NT	negative	3	Dog (8)	8 (4.3)


*^a^One isolate with scm type 10 (allele 10.2) was non-typeable in the emm type PCR. NT, non-typeable*.

*^b^PCR using primers all_canis-fwd/rev previously described (Fulde et al., 2011).*

Most isolates of a given ST had the same *scm* type, resulting in ST being a good predictor of *scm* type (AW_ST→_*_scm_*_type_ ± 95% CI, 0.966 ± 0.031) (Supplementary Figure 2). In contrast, *scm* type was a poor predictor of ST (AW*_scm_*_type→ST_ ± 95% CI, 0.374 ± 0.104), although the same *scm* type was often found among isolates belonging to the same CC_SLV_ (AW_CC→_*_scm_*_type_ ± 95% CI, 0.949 ± 0.034) with a weaker correspondence in the reverse direction (AW*_scm_*_type→CC_ ± 95% CI, 0.793 ± 0.072). The following were the main lineages found ( > 10 isolates):CC9/*scm1* (*n* = 71, 37.8%), CC13/*scm2* (*n* = 28, 14.9%), CC2/*scm3* (*n* = 18, 9.6%), CC1/*scm8* (*n* = 17, 9.0%), and CC16/*scm2* (*n* = 11, 5.9%) (Supplementary Table 3).

### Diversity of Predicted SCM Proteins

Translation of the 41 *scm* alleles resulted in 40 distinct predicted proteins (alleles *scm1.1* and *scm1.7* resulted in the same amino acid sequence). These included three *scm* type 1 isolates with premature stop codons, generating ORFs with 594 bp (*scm1.16* allele) and 192 bp (*scm1.17* and *scm1.18* alleles). In the later two isolates, an alternative reading frame was identified starting 148bp downstream of the usual start-site, resulting in 1164 bp long ORFs (387 amino acids), in which no signal peptide could be identified. This was also the case of one isolate with an *scm* type 2 allele (*scm2.2*) which started 48 bp downstream of the start-site of alleles of the same type. In all other SCM variants the presence of a signal peptide was predicted by SignalP 4.1, with three possible cleavage sites found for different proteins, namely between amino acids 34 and 35 (types 1 to 3 and 5 to 7), amino acids 32 and 33 (type 4), and 41 and 42 (types 8 to 12). The SCM proteins of group I (SCM types 1 to 7) and group II (SCM types 8 to 12) had less than 33% amino acid identity to each other, with high sequence identity (> 90%) being observable only in the last 50 amino acids of the C-terminus of the proteins, which corresponds to the LPXTG cell wall anchor domain.

**FIGURE 3:**
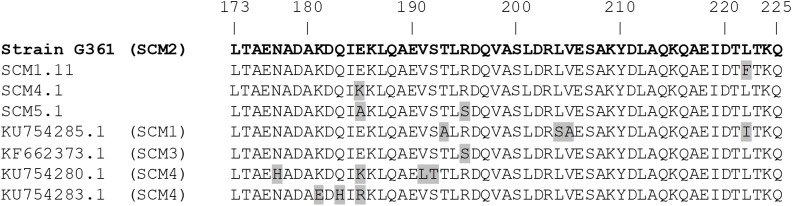
Alignment of predicted amino acid sequences of IgG binding regions in SCM group I proteins. The IgG binding region derived from isolate G361 comprising amino acids 173 to 225 of the mature protein (Bergmann et al., 2017) was used as reference and corresponds to the consensus of predicted SCM group I proteins. Representative alleles of sequences found in our study are indicated by their numbers and sequences found elsewhere by their GenBank numbers.

Most of the sequence variability among proteins predicted from group I *scm* alleles (SCM types 1 to 7) occurred in a region encompassing approximately the first 100 amino acids of the mature SCM protein, corresponding to the hypervariable *N*-terminal portion of the protein (Supplementary Figure 3). The remaining portion of the sequence was highly conserved, with amino acid sequence identity higher than 98% for most of the group I SCM variants newly identified in our study, although more divergent sequences (75% amino acid sequence identity) were present among those previously reported (Timoney et al., 2017). The IgG-binding region of SCM, corresponding to amino acids 173 to 225 of the mature protein of isolate G361 (Bergmann et al., 2017), is located in this conserved region and showed minimal sequence variation. An amino acid sequence identical to that of isolate G361 was found in 132 of the 142 isolates representing group I SCM, with only 4 alleles (9 isolates), SCM1.11 (L222F), SCM1.17 (L222F), SCM4.1 (E185K), and SCM5.1 (E185A and R195S), presenting amino acid changes in this region of the protein (Figure 3). Up to 4 amino acid differences were observed in the SCM protein derived from sequences described elsewhere (Timoney et al., 2017), but not found in this study. The predicted sequence found in the genome of strain FSL Z3-227 had a deletion of a stretch of 49 amino acids (amino acids 191 to 239 of the G361 protein), resulting in the absence of the IgG-binding region. This SCM protein and the SCM1.16 variant (noted above for possessing a premature stop codon), were the only group I SCM variants in which this region was not present.

In group II *scm* alleles (SCM types 8 to 12) the hypervariable portion of the protein was larger (up to 280 amino acids in SCM type 11) and we could find no conserved IgG-binding region (Supplementary Figure 3). Despite the low sequence identity to group I SCM types, the presence of an *N*-terminal signal peptide, a C-terminal LPXTG cell wall anchor domain (M protein-type) and a two-stranded coiled-coil conformation was predicted in all group II SCM variants, consistent with these two groups being functional homologs.

## Discussion

The recognized pathogenic role of *S. canis* in companion animals, cows and more recently humans, has prompted an increasing number of studies probing the genetic properties of *S. canis* isolates recovered from these hosts (Richards et al., 2012; Pinho et al., 2013; Taniyama et al., 2017; Timoney et al., 2017). Isolation from wild animal species has been reported sporadically and genotyping of these isolates has been rare, although the range of distinct hosts from which it has been recovered (Castinel et al., 2007; Hariharan et al., 2011; Nikolaisen et al., 2017; Seguel et al., 2018) suggests that a broad host tropism is one of the hallmarks of *S. canis*.

Our study describes the first MLST characterization of *S. canis* wildlife isolates. Most of the wildlife isolates were associated with STs or CC_SLV_ found among isolates recovered from companion animals, livestock and humans, indicating that they are not genetically distinct and confirming previous observations that some *S. canis* lineages are found in many animal species (Richards et al., 2012; Pinho et al., 2013). A prime example is the CC9 lineage, comprising more than one third of the isolates analyzed (37.8%), which has been previously shown to dominate among companion animals (Pinho et al., 2013), humans (Pinho et al., 2013) and cows (Richards et al., 2012) from distinct geographic regions. We found CC9 to predominate in seal and otter isolates and showed that this genetic lineage is associated with *scm* type 1, also the most common *scm* type in our study. Taken together, our results indicate that CC9/*scm1* is the most important *S. canis* genetic lineage for many distinct animal hosts.

Although isolation of *S. canis* from pinnipeds has been documented (Castinel et al., 2007; Seguel et al., 2018), previous studies characterizing beta-hemolytic streptococci from marine mammals from the North and Baltic Seas identified *Streptococcus phocae, Streptococcus equi* subsp. *zooepidemicus* and *Streptococcus dysgalactiae*, but not *S. canis* (Swenshon et al., 1998; Vossen et al., 2004; Akineden et al., 2007). These species were also recovered from seal carcasses in our study. While *S. phocae* predominated, similar isolation rates were observed for *S. canis* and the two other species (data not shown). Despite being collected over a period of 20 years (1993 to 2012), all seal isolates represented CC9. This uniformity seems not to be attributable to the restricted geographical distribution of the seals (most were isolated from seals found along the Scottish Coast), since it was not observed among isolates from otters and the dog isolates, also exclusively recovered in Scotland, which showed a diversity comparable to the one of *S. canis* isolates recovered in other European countries (Pinho et al., 2013). Our results are similar to the ones reported for *S. equi* subsp. *zooepidemicus* (Akineden et al., 2007) and *S. dysgalactiae* (Swenshon et al., 1998) from marine mammals, in which dominance of a single clone has been noted. It is conceivable that the wide host distribution of CC9 may facilitate transmission, perhaps also through environmental sources, or that this lineage has specific factors allowing infection and persistent in multiple host populations.

The *S. canis* M-like protein SCM has been the subject of recent studies focusing on its function (Yang et al., 2010; Fulde et al., 2011, 2013; Bergmann et al., 2017) or using it to type *S. canis* (Taniyama et al., 2017; Timoney et al., 2017). Although the *scm* locus was identified in the first *S. canis* genome available (Richards et al., 2012), the correspondence of *scm* and the *emm* types found in *S. canis* (Ahmad et al., 2009; Pinho et al., 2013) had not been further explored. The percentage of *scm*-typeable isolates in our collection (75%) using the previously proposed method was comparable to the one previously reported (68%) (Fulde et al., 2011), while an *emm* type was obtained for a minority of isolates, also in line with previous studies reporting the inability of *emm-*typing most *S. canis* isolates (Whatmore et al., 2001; Ahmad et al., 2009; Pinho et al., 2013). The genome arrangement around the *stG1389 emm-*like gene resembled the genomic context of the *emm* gene in SDE and was identical to the genomic context of the *scm* gene in the three previously available *S. canis* genomes (all *scm*-typeable isolates). We expanded this observation to all *S. canis* isolates in our collection by using a novel PCR targeting the *scm* flanking genes, which showed that an *scm* gene was present in all *S. canis* isolates tested. Moreover, identical *scm* sequences were obtained by both the novel and *emm* typing PCRs for *stG1389*, *stG1451*, and *stG663* isolates, confirming that these 3 *scm* genes are the only ones that have been designated as *emm* types within the CDC *emm* type database^10^ because these are detected using the primer pair that has been most often used for *emm* typing (Ahmad et al., 2009; Pinho et al., 2013). Taken together, our results confirmed that the *S. canis scm* locus corresponds to the *emm* locus present in SDE and GAS and that this locus is universally present among *S. canis* isolates, contrary to what had been reported.

The new PCR protocol described in this study is the first allowing assignment of an *scm*/*emm* type to all *S. canis* isolates tested. The new method improves *emm* typing, the most widely used typing method in SDE and GAS, by allowing its universal application in *S. canis* typing. In *emm* typing, *emm* types are defined based on less than 92% identity within the first 30 codons encoding the mature M protein,^11^ corresponding to the *N*-terminal hypervariable terminus of the protein. We confirmed that the *scm* types defined in our study comply with the criteria used in *emm* typing based on the NJ tree (Figure 2). Since it is not necessary to sequence the entire *scm* gene to determine the *scm* type, we propose to use primers Sc_Mprot_F2 and Sc_Mprot_R2 (Supplementary Table 2) for amplification (fragment size ranging from approximately 1 to 1.4 kb) and of Sc_Mprot_F2 for sequencing to define *scm* types, similarly to the *emm* typing protocol.

The current study expands the known diversity of SCM proteins from 4 (Timoney et al., 2017) to 12 different types and shows that SCM proteins of *S. canis* can be divided into two major groups. SCM types belonging to group I dominate among *S. canis* from all hosts and what is known about SCM function concerns this group of proteins since it corresponds to the previously *scm*-typeable isolates. SCM is thought to have an anti-phagocytic role by interacting with the conserved Fc domain of IgG in a non-opsonic manner (Bergmann et al., 2017). We found the IgG-binding region, consisting of 52 amino acids in the central part of the mature SCM (Bergmann et al., 2017), to be highly conserved among group I SCM types, in agreement with previous observations (Bergmann et al., 2017). Plasminogen has also been identified as an SCM ligand (Fulde et al., 2011, 2013) and the plasminogen-binding region in SCM has been identified within the first 214 amino acids of the mature protein (Fulde et al., 2011). This region encompasses the hypervariable portion of SCM and we found substantial amino acid variation between each of the individual SCM types. Thus, inferences on plasminogen binding ability of the different SCM protein variants based solely on their sequences may be unreliable, and it is conceivable that differences in plasminogen-binding ability may occur even within group I SCM types.

Our study extended previous observations by showing that *S. canis* isolates from wildlife were not genetically distinct from those found among animals with close contact with humans and humans themselves. This is compatible with frequent transmission between species which could have important consequences when considering the zoonotic potential of *S. canis*. We found that the *scm* gene is universally present in *S. canis* and identified divergent SCM proteins among isolates previously reported as *scm*-negative. The function of these group II SCM proteins is unclear since they present limited sequence similarity with those of group I along almost the full protein length and the IgG binding domain, typical of group I SCM types, is absent. Nevertheless, group II SCM proteins present structural features typical of M-like proteins indicating these may also interact with host factors and have a role in pathogenesis. Since there were no apparent differences in host distribution or infection type between isolates carrying either group I or group II SCM variants, we have no clues as to the potential phenotypic differences conferred by the presence of each of these variants. It was recently suggested a novel *emm*-cluster-based system to classify the M-proteins of GAS (Sanderson-Smith et al., 2014). The M-proteins encoded by each of the two major clusters identified among GAS *emm* (clades *X* and *Y*), shared few interactions with host factors (Sanderson-Smith et al., 2014) and this seems to be also the case with SCM’s two groups. Despite these similarities, both clades *X* and *Y* of GAS are outgroups of the *S. canis scm* gene (data not shown), not indicating a recent relationship of any of the *S. canis* groups with any of the GAS clades. Further studies are necessary to identify the possible host interaction partners of group II SCM to clarify the role of these proteins in pathogenesis.

## Data Availability

The datasets generated for this study can be found in *S. canis* PubMLST database, GenBank and European Nucleotide Archive (ENA). All MLST data, including novel alleles and STs, were submitted to the *S. canis* PubMLST database (http://pubmlst.org/scanis/). *scm* sequences were submitted to GenBank under accession numbers MH996642 to MH996682. The raw sequencing reads and the annotated assembled genome of *S. canis* isolate FMV2238.02 are available through the European Nucleotide Archive (ENA) under the study accession number PRJEB29117.

## Author Contributions

MP performed the experiments. PGSSI, GF, CP, JB and TK collected data. MP, MM and MR analyzed and interpreted the data. MP, GF, JM-C and MR were involved in the conception and design of the study. MP, GF, CP, MM, JM-C and MR were involved in drafting the manuscript and revising it critically for important intellectual content.

## Conflict of Interest Statement

JM-C has received research grants administered through his university and received honoraria for serving on the speakers bureaus of Pfizer and Merck Sharp, and Dohme. MR has received honoraria for serving on the speakers bureau of Pfizer and for consulting for GlaxoSmithKline and Merck Sharp and Dohme. The remaining authors declare that the research was conducted in the absence of any commercial or financial relationships that could be construed as a potential conflict of interest.

## Footnotes

1 http://pubmlst.org/scanis/

2 https://github.com/B-UMMI/INNUca

3 https://hub.docker.com/r/ummidock/innuca/

4 https://www.bioinformatics.babraham.ac.uk/projects/fastqc/

5 https://hub.docker.com/r/ummidock/prokka/

6 https://www.ncbi.nlm.nih.gov/genome/11108

7 http://www.cbs.dtu.dk/services/SignalP/

8 https://www.ebi.ac.uk/interpro/

9 http://www.comparingpartitions.info

10 https://www.cdc.gov/streplab/groupa-strep/emm-typing-protocol.html

11 https://www.cdc.gov/streplab/groupa-strep/emm-background.html
