# Supplementary material for: Streptococcus canis Are a Single Population Infecting Multiple Animal Hosts Despite the Diversity of the Universally Present M-Like Protein SCM
Source: Front Microbiol. 2019 Mar 29;10:631. doi: 10.3389/fmicb.2019.00631 (PMC6450190; doi:10.3389/fmicb.2019.00631)
Supplement: Supplementary file 1 [file Data_Sheet_1.PDF]

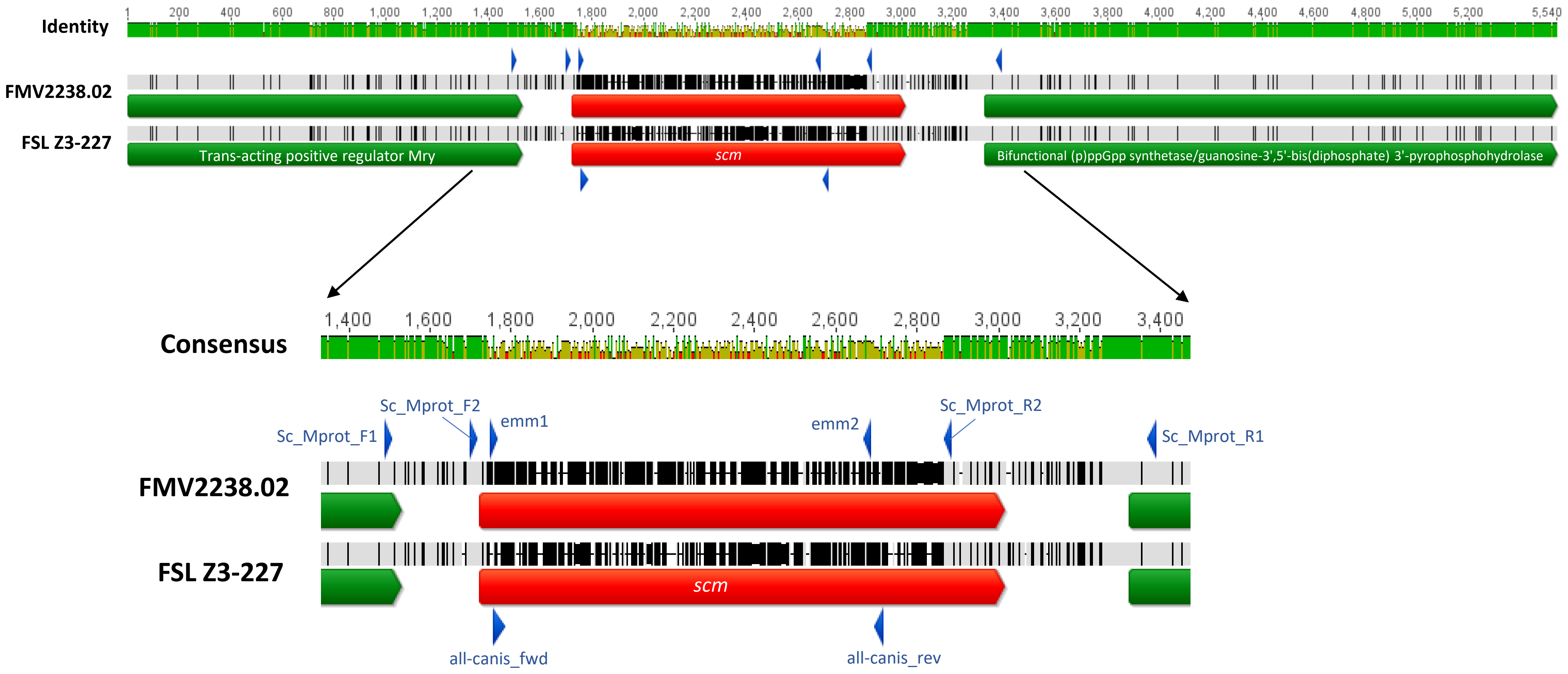

Supplementary Figure 1. Sequence alignment of the *scm* regions of *S. canis* isolates FMV2238.02 and FSL Z3-227. The *scm* gene is colored in red. Genes flanking the *scm* gene in *S. canis* are colored in green and annotated with the names of isolate FSL Z3-227 (GenBank accession number NZ_AIDX01000001). The primer binding sites for the primer pairs used in this study are shown in blue. Numbers above the identity diagram indicate base pairs. Geneious version 8.1.9 (Biomatters) was used to align the sequences.
