# Supplementary material for: Streptococcus canis Are a Single Population Infecting Multiple Animal Hosts Despite the Diversity of the Universally Present M-Like Protein SCM
Source: Front Microbiol. 2019 Mar 29;10:631. doi: 10.3389/fmicb.2019.00631 (PMC6450190; doi:10.3389/fmicb.2019.00631)
Supplement: Supplementary file 2 [file Data_Sheet_2.PDF]

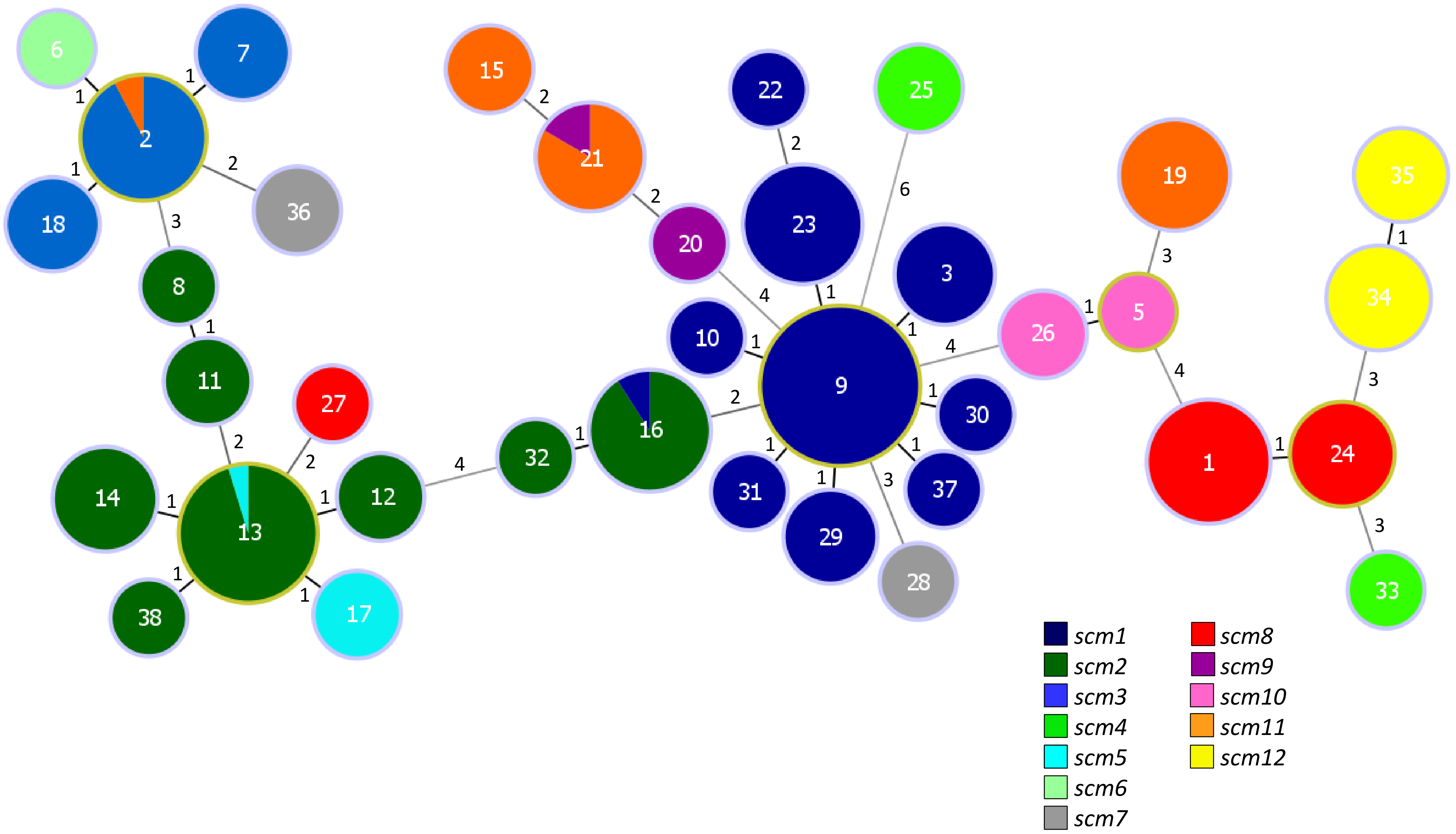

Supplementary Figure 2. goeBURST diagram of *S. canis* isolates with different *scm* types. The diagram includes the 188 isolates studied plus the 3 *S. canis* isolates with public available genomes (from witch MLST data was extracted). Numbers inside circles identify the ST and numbers near straight lines connecting circles indicate the distance level between two given STs. The size of each circle is proportional to the number of isolates with that particular ST on a logarithmic scale. The number of isolates with the same characteristic is proportional to the respective color. Putative CC founders are identified by a light green circle and correspond to the STs with the higher number of SLVs.
