# Supplementary material for: Streptococcus canis Are a Single Population Infecting Multiple Animal Hosts Despite the Diversity of the Universally Present M-Like Protein SCM
Source: Front Microbiol. 2019 Mar 29;10:631. doi: 10.3389/fmicb.2019.00631 (PMC6450190; doi:10.3389/fmicb.2019.00631)
Supplement: Supplementary file 3 [file Data_Sheet_3.PDF]

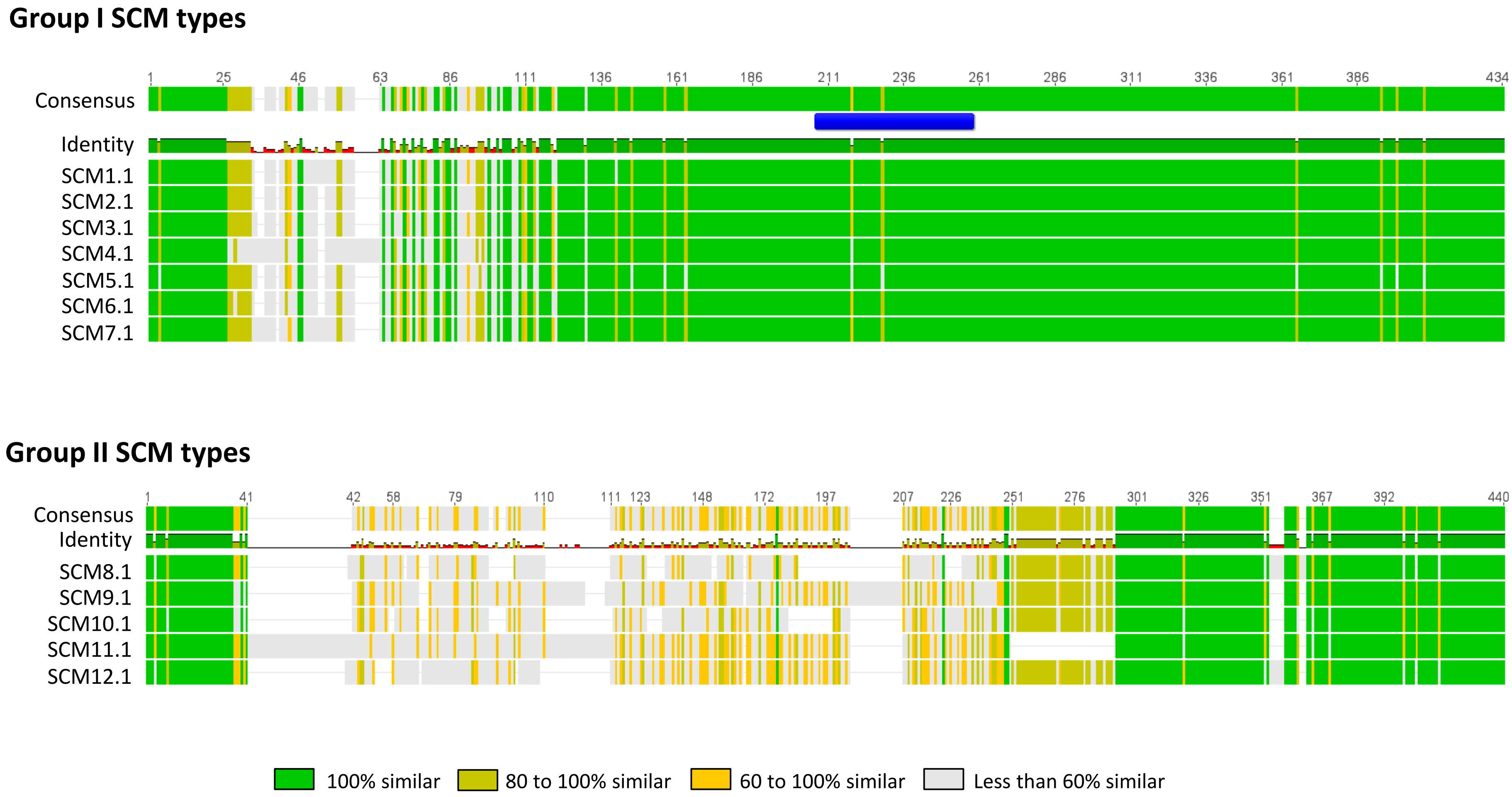

Supplementary Figure 3. Amino acid sequence alignments of representatives of SCM types belonging to group I or group II SCM.
The most common SCM variant of each SCM type was included in the alignment. The position of the IgG binding site present in group I SCM types is indicated in blue. Numbers above the consensus diagram indicate amino acids. Geneious version 8.1.9 (Biomatters) was used to align the sequences.
